# Lessons for Uptake and Engagement of a Smartphone App (SURE Recovery) for People in Recovery From Alcohol and Other Drug Problems: Interview Study of App Users

**DOI:** 10.2196/33038

**Published:** 2022-03-08

**Authors:** Joanne Neale, Alice May Bowen

**Affiliations:** 1 National Addiction Centre King's College London London United Kingdom

**Keywords:** apps, digital literacy, mHealth, substance use, recovery, qualitative, mobile phone

## Abstract

**Background:**

Mobile health apps promoting health and well-being have substantial potential but low uptake and engagement. Barriers common to addiction treatment app uptake and engagement include poor access to mobile technology, Wi-Fi, or mobile data, plus low motivation among non–treatment-seeking users to cut down or quit. Working with people who used substances, we had previously designed and published an app to support recovery from alcohol and other drug problems. The app, which is available for free from the Apple App Store and Google Play, is called *SURE Recovery.*

**Objective:**

The aim of this paper is to undertake a qualitative study to ascertain end users’ views and experiences of the SURE Recovery app, including how it might be improved, and present the findings on uptake and engagement to assist other researchers and app developers working on similar apps for people experiencing alcohol and other drug problems.

**Methods:**

Semistructured telephone interviews were conducted with 20 people (n=12, 60%, men and n=8, 40%, women aged 25-63 years; all identifying as White British) who had varied patterns of using the app. The audio recordings were transcribed, and the data were coded and analyzed through Iterative Categorization.

**Results:**

Analyses identified three main factors relevant to *uptake* (discoverability of the app, personal relevance, and expectations and motivations) and three main factors relevant to *engagement* (the appeal and relevance of specific features, perceived benefits, and the need for improvements). The findings on uptake and engagement were largely consistent with our own earlier developmental work and with other published literature. However, we additionally found that uptake was strongly affected by first impressions, including trust and personal recommendations; that users were attracted to the app by their need for support and curiosity but had relatively modest expectations; that engagement increased if the app made users feel positive; and that people were unlikely to download, or engage with, the app if they could not relate to, or identify with, aspects of its content.

**Conclusions:**

Incorporating end-user views into app design and having a network of supportive partners (ie, credible organizations and individuals who will champion the app) seem to increase uptake and engagement among people experiencing alcohol and other drug problems. Although better digital literacy and access to devices and mobile data are needed if addiction recovery apps are to reach their full potential, we should not evaluate them based only on observable changes in substance use behaviors. How using an app makes a person feel is more transient and difficult to quantify but also relevant to uptake and engagement.

## Introduction

### Background

The use of mobile health (mHealth) apps is increasingly common among clinical and nonclinical populations, including people who use substances. With the ownership of smartphones, tablets, and wearable devices growing and access to wireless networks expanding, the number of apps relating to substance use has proliferated and downloads of the most popular apps have risen [1,2]. Alongside their potential reach, mHealth apps are convenient (they can be accessed anytime and anywhere), are low cost, and can overcome some of the barriers to accessing standard treatment (such as strict appointment times, lengthy distances to travel, concerns regarding childcare, and stigma) [2-5]. Apps can also facilitate personalized (*tailored*) support; offer opportunities for real-time relapse prevention, treatment, and aftercare [6-8]; and are accessible to people already in treatment as well as those not currently accessing services [7].

Addiction-related apps commonly include blood alcohol content calculators, service finders, other information and resources, games to distract from cravings, strategies to increase motivation, functions to enhance social support, and tools to monitor progress [1,2,8]. Accordingly, they offer valuable opportunities for diagnostics, measurement, treatment, and recovery [1]. Evidence suggests that users particularly like behavior tracking and remote access to advice and information [8]. In addition, they appreciate the portability of apps, their discretion (given the stigma around addiction), and the fact that they tend to be free or low cost [2,8]. However, alcohol-related apps are often designed for entertainment or to promote, rather than reduce, alcohol use [7], and cannabis apps tend to be for informational or recreational purposes [9]. Furthermore, treatment-oriented apps predominantly focus on tobacco and alcohol, rather than illicit drugs, and have mostly been developed in the United States, potentially limiting their relevance internationally [8-11].

Importantly, apps promoting health and well-being also tend to suffer from low uptake and engagement [11]. Uptake is the act of downloading and installing a smartphone app, whereas engagement refers to both the extent (eg, amount, frequency, duration, and depth) of use and the user’s personal experience as characterized by, for example, their attention, interest, or mood [11,12]. Barriers to addiction treatment app uptake and engagement include poor access to mobile technology, Wi-Fi, or mobile data [13,14] as well as low motivation among non–treatment-seeking users to cut down or quit [9]. Compounding these limitations, evidence for the effectiveness of smartphone apps in addressing alcohol and other drug problems is weak [2,7,8,15]. This may be related to the poor quality of much app content; for example, apps rarely include empirically based behavior change techniques (other than self-monitoring) [2,9,15,16] and app developers often lack personal or clinical experience of addiction [8].

Before developing a new recovery app to help people reduce or cease their alcohol and other drug use and improve their quality of life, it seems sensible to ask whether there is a genuine demand. This question is likely to be especially important if people who use substances do not want to change their behavior, have limited resources or complex needs, and experience high levels of digital exclusion. Although a candid answer may be that demand is likely to be weak, it still seems wrong to perpetuate inequalities and lack of choice by failing to offer digital options for those who might be interested or benefit. A better alternative would be to work with the target population to develop an app that might support at least some people in addressing their substance use while also seeking to learn from the process and results. It is on this basis that we developed the SURE Recovery app [17].

People with personal experience of addiction had asked us to convert our two validated pen and paper measures, the Substance Use Recovery Evaluator (*SURE*; a 21-item measure of addiction recovery) [18] and the Substance Use Sleep Scale (*SUSS*; a 23-item measure of sleep problems experienced by people using substances) [19] into an app that they could complete on their mobile phones and tablet computers. They explained that they wanted to record and refer back to their SURE and SUSS scores, and they also expressed a desire for personalized feedback. Further discussions suggested that they would like to see the 2 measures supplemented with other features that might promote recovery from alcohol and other drug problems. Both SURE and SUSS had been developed collaboratively with people who had experience of addiction, and we continued this joint working by adopting a co-design approach when developing the SURE Recovery app.

Co-design involves *end users* throughout the design process as active partners [20], providing people whose lives might be affected by a problem with a voice in its solution [21,22]. Evidence suggests that the inclusion of end users in the early stages of the design process leads to better outcomes and more benefits compared with ideas developed by designers alone [23]. Our co-design approach was completed following the Double Diamond design process, which is a framework widely used in the design industry. This involves four distinct phases—(1) discover, (2) define, (3) develop, and (4) deliver—that are repeated in iterative cycles to ensure that end-user feedback is incorporated throughout [24]. To this end, we conducted interviews, focus groups, review meetings, and testing sessions with nearly 50 people in recovery or actively using substances. In addition, our team comprised people with personal experience of alcohol and other drug problems, clinicians (addiction psychologists and psychiatrists), and academics (social scientists and statisticians).

During the *discover* phase of our work, interview and focus group participants explained that they valued different types of formal and informal support, enjoyed connecting with others in similar situations, appreciated being busy and distracted, felt that keeping a log of their recovery was helpful, and wanted advice on the types of support available. When asked to comment on the design and content of other apps, they expressed preference for a clear layout, bright colors, simplicity, tracking features, inspirational quotations, nonjudgmental and supportive language, an opportunity to share artwork, and the ability to connect with others. In contrast, they disliked apps that seemed *busy* or *crowded*, had too much text, contained advertisements, or looked *technical*. From this feedback, a long list of potential app features was created. This included information and advice; a directory of services; opportunities to meet or share personal stories, experiences, advice, and artwork; tracking (progress, mood, or problems); a way to be reminded of the app; encouragement and motivation; and sleep tracking. Following discussions during the *define* phase, the team narrowed the options down to 6 features that were viable within the project budget and time frame, plus a set of optional research questions covering basic demographics, substance use, and treatment-related topics.

### App Features

The six features included in the SURE Recovery app are as follows:

1. A recovery tracker (this allows people to monitor their own recovery through SURE and receive personalized feedback and a score that can be viewed on a graph)
2. A sleep tracker (this works in a similar way to the recovery tracker, enabling people to monitor their sleep through SUSS and receive personalized feedback, a score, and a graph)
3. Artwork (app users can submit their artwork for potential display in the banner of the app home screen)
4. Diary (a private space where people can record their thoughts and feelings)
5. Naloxone (an instructional video on how to use the life-saving medication naloxone in the event of an opioid overdose, plus informational resources and a knowledge tracker to measure overdose management competency)
6. Resources (free access to a book,*The Everyday Lives of Recovering Heroin Users*, which is based on the lived experiences of people in recovery) [25]

After much reflection, we did not include a social feature, where app users could *chat* and share experiences and advice, because the team did not have adequate resources to monitor the chat in a way that would ensure app user safety at all times.

The SURE Recovery app has been available to download for free from the Apple App Store and Google Play since October 2019 (>2200 downloads by May 31, 2021). It was updated with a temporary COVID-19 pop-up feature (comprising COVID-19 resources, information, and a new research question) in April 2020. In March 2021, the temporary COVID-19 feature was replaced by a more permanent and dynamic *hot topic of the month* feature (allowing information on a contemporary relevant issue to be displayed, an associated research question to be asked, and key findings from any responses received to be posted back into the body of the app). Given the importance of understanding what end users thought of SURE Recovery, including their views on whether and how it might be improved going forward, 2 members of the SURE App team (JN and AMB) also conducted a qualitative study. The aim of this paper is to present our findings on uptake and engagement to assist others developing similar apps for people experiencing alcohol and other drug problems.

## Methods

### Ethics Approval

Ethical approval for the qualitative study was received from the research ethics committee of King’s College London (HR-19/20-17338). 

### Overview

Data were generated through semistructured telephone interviews conducted with 20 people who had downloaded the SURE Recovery app. When signing up for the app, all users are provided with a link to a web-based information sheet and asked if they are willing to share their anonymized data for quantitative research. If they agree, they are given the option to consent within the app. App users are next asked if they would be willing to be contacted by a researcher to participate in further research relating to SURE Recovery. Those who agreed to both share their data and be contacted for further research (N=620) were entered into a pool of potential participants for the qualitative study. For pragmatic reasons (the cost of international telephone calls, time differences, increased likelihood of poor telephone reception, and language differences), of the 620 respondents, we excluded 241 (38.9%) who were based outside the United Kingdom, leaving 379 (61.1%) potential participants. From these, we sampled purposively to include people who had used the app once or twice only, occasionally, and frequently. As a secondary strategy, and to be as inclusive of views as possible, we also endeavored to sample people with a mix of demographic, substance use, and treatment characteristics.

Author AMB first contacted potential participants through the email address they had used when registering for the app. In total, 107 app users were contacted in this way over the course of 10 months (May 2020 to February 2021). The email sent contained basic information regarding the qualitative study and invited the recipient to respond with a telephone number if they wanted to hear more. A maximum of 3 invitation emails were sent to each person. People who responded positively (24/107, 22.4%) were then emailed the study information sheet and consent form and asked to select a time when they could be interviewed. An additional telephone call was offered to anyone who wanted to know more about the study before deciding whether to participate. At this point, of the 24 participants, 4 (17%) withdrew their interest, whereas 20 (83%) agreed to continue. AMB conducted all interviews by telephone, securing verbal consent before each interview started. Although the target number of participants had been 30, recruitment ceased after 107 app users had been contacted and 20 interviews had been completed. This was because both authors believed that data saturation had been achieved: comments regarding the app from new interviews were largely repeating comments from earlier interviews and no new themes or topics seemed to be emerging [26].

All interviews were audio recorded and followed a topic guide that covered the participant’s background (general life circumstances, health, education, employment, substance use, and treatment history), initiation to SURE Recovery (how the participant had first heard about SURE Recovery, their expectations, motivations, goals, and reasons for downloading the app), use of SURE Recovery (frequency; duration; cessation; when, where, and how the app was used; barriers to use; and features most used), positive views of SURE Recovery (features liked and any benefits of use), negative views of SURE Recovery (features disliked and any negative consequences of use), and potential improvements to SURE Recovery (suggested improvements, strategies for overcoming barriers to use, and ideas for new features). Interviews lasted 18-73 minutes, and participants were paid £20 (US $27.20) as compensation for their time.

### Data Analyses

Data analyses followed the stages of Iterative Categorization [27,28]. To begin with, the audio files were transcribed verbatim by a professional transcription service and the transcriptions were uploaded to the software data management program MAXQDA (version 2018.2; VERBI Software GmbH) [29]. Next, both authors jointly devised a simple coding frame that mirrored the interview topic guide. Subsequently, AMB indexed all transcribed text to one or more of the codes and exported the indexed data from the software program into Microsoft Word documents (1 Word document per code). Each Word document was then reviewed line by line (either by AMB or by JN) to identify patterns and themes in the data. To this end, all indexed text was summarized into bullet points, and the bullet points were iteratively grouped into themes and categories that were in turn summarized (1 summary per code). Next, JN combined the summaries from each code into 1 main findings document that provided an overarching descriptive account of the participants’ views. JN then systematically reviewed the main findings document for material relating to SURE Recovery uptake and engagement before AMB checked and confirmed the findings.

## Results

### Participant Characteristics

Table 1 presents basic data relating to all people who downloaded the app between October 1, 2019, and May 31, 2021; consented to share their data; and consented to be contacted for further research (N=620); and all people who downloaded the app between October 1, 2019, and May 31, 2021; consented to share their data; consented to be contacted for further research; and were based in the United Kingdom (379/620, 61.1%); as well as all participants who contributed a qualitative interview (20/379, 5.3%). These data are provided to contextualize the qualitative study participants within the wider body of app users. Table 1 suggests that those participating in the qualitative interviews may have been more likely to have ever had a problem with use of opioids or alcohol, to have attended mutual aid meetings or peer support groups in the last week, and to be in paid work than other app users. In contrast, they were potentially less likely to have used substances or to have been in formal treatment in the last week. This might simply reflect the fact that individuals who are more stable in recovery are more willing and able to participate in a qualitative telephone interview than those who are still regularly using substances and in formal treatment.

As seen in Table 1, of the 20 people who participated in a qualitative interview, 12 (60%) were men and 8 (40%) were women. They had a mean age of 43 (range 25-63) years, and all were White British. The qualitative interviews provided additional and more comprehensive demographic information and drug use data about the study participants. When interviewed, 8 (40%) said that they were in paid employment (of these 8 participants, 5, 63%, said that they worked in the drug treatment sector); 10 (50%) reported that their substance of choice was alcohol and 10 (50%) said that their substance of choice was another psychoactive drug; and 8 (40%) had ever injected a drug. Although all 20 (100%) participants identified as ever having had a problem with alcohol or other drugs, 13 (65%) said that they had not used any substances in the last month and 6 (30%) said that they were neither currently receiving formal treatment nor attending any mutual aid or peer support groups.

**Table 1:** SURE Recovery app user characteristics (October 1, 2019, to May 31, 2021).

Characteristics		All users who consented to share their data and be contacted for further research (N=620)	All users based in the United Kingdom who consented to share their data and be contacted for further research (n=379)	Users participating in a qualitative interview (n=20)
**Gender, n (%)**				
	Male	269 (43.4)	192 (50.7)	12 (60)
	Female	333 (53.7)	182 (48)	8 (40)
	Other	7 (1.1)	1 (0.3)	0 (0)
	Prefer not to say	11 (1.8)	4 (1)	0 (0)
**Age (years)**				
	Value, mean (SD)	41 (11)	42 (10.7)	43 (10.5)
	Missing, n (%)	82 (13.2)	32 (8.4)	0 (0)
Ethnicity^a^ (White British), n (%)		N/A^b^	N/A	20 (100)
App users who completed optional questions on first-ever use of the app^c^, n (%)		308 (49.7)	184 (48.5)	6 (30)
**Participated in paid employment during the last week, n (%)**				
	Yes	176 (57.1)	110 (59.8)	5 (83.3)
	No	132 (42.9)	74 (40.2)	1 (16.7)
**Ever had a problem with use of heroin or other opiates, n (%)**				
	Yes	89 (28.9)	43 (23.4)	3 (50)
	No	219 (71.1)	141 (76.6)	3 (50)
**Ever had a problem with use of alcohol, n (%)**				
	Yes	203 (65.9)	136 (73.9)	5 (83.3)
	No	105 (34.1)	48 (26.1)	1 (16.7)
**Any substance use in the last week, n (%)**				
	Yes	229 (74.4)	129 (70.1)	1 (16.7)
	No	79 (25.6)	55 (29.9)	5 (83.3)
**Contact with community drug and alcohol treatment services in the last week, n (%)**				
	Yes	103 (33.4)	65 (35.3)	1 (16.7)
	No	205 (66.6)	119 (64.7)	5 (83.3)
**Attended mutual aid meetings or a peer support group in the last week, n (%)**				
	Yes	114 (37)	72 (39.1)	3 (50)
	No	194 (63)	112 (60.9)	3 (50)
**In residential treatment during the last week, n (%)**				
	Yes	15 (4.9)	8 (4.3)	0 (0)
	No	293 (95.1)	176 (95.7)	6 (100)

^a^Apple does not permit developers to require personal information that is not directly relevant to the app’s core functionality at registration. We decided to not include an optional ethnicity question, given the number of sensitive optional questions regarding substance use already being asked and concerns that many potential users may consider an ethnicity question irrelevant or be frustrated by a long scroll list that may make finding their own ethnicity difficult. The lack of ethnicity data has resulted in a limitation in our analyses, which we discuss further in the *Limitations* section.

^b^N/A: not applicable.

^c^SURE Recovery users can return and complete optional questions at any time when using the app. For consistency, only data entered by users on their first occasion of using the app are reported. This means that the number of responses to various questions in the table is less than the number of app users (n=308, n=184, and n=6 rather than N=620, n=379, and n=20).

### Participants’ Use of SURE Recovery

Consistent with our recruitment strategy, use of the app by our qualitative study participants varied greatly. Thus, we interviewed people who had recently downloaded the app but had not yet started to use it; had used it once or twice and then stopped; had used it frequently initially but were now using it less; were using it daily; and were using it occasionally. Further details regarding the participants’ use of SURE Recovery are shown in Table 2.

Turning to our main qualitative analyses, we identified 3 key factors relevant to decisions to download and install the app, that is, *uptake*. These were (1) discoverability of the app, (2) personal relevance, and (3) expectations and motivations. In addition, we found that 3 key factors affected use and experiences, that is, *engagement*. These were (1) the appeal and relevance of specific features, (2) perceived benefits, and (3) the need for improvements. We next present our findings using pseudonymized quotations to illustrate salient points.

**Table 2:** Participants’ use of SURE Recovery (N=20)^a^.

Type of SURE Recovery use			Values, n (%)	
**Current use**				
	Yes		11 (55)	
	No		6 (30)	
	About to start or restart		3 (15)	
**Frequency of current use**				
		Daily	2 (10)	
		Weekly	6 (30)	
		Monthly	3 (15)	
		Unknown	9 (45)	
**Data transfer source**				
		Wi-Fi	9 (45)	
		Cellular	2 (10)	
		Wi-Fi and cellular	8 (40)	
		Unknown	1 (5)	
**Device used**				
		Android phone	6 (30)	
		iPhone	6 (30)	
		iPhone and Android tablet	1 (5)	
		Unknown	7 (35)	

^a^Comparable data are not available for app users who did not participate in a qualitative interview.

### Uptake

#### Discoverability of the App

Knowing how participants first heard about SURE Recovery provides potentially important information on how addiction-related apps are *discovered* and thus how they might be introduced or even *advertised* to potential users. Most study participants had first learned about SURE Recovery either through a key worker, support worker, or professional who was working with them or from browsing or searching for addiction-related information and support on the web. A few participants who were employed as recovery workers within the addiction treatment sector said that they had come across the app during the course of their work. In addition, 1 (5%) had read about the app in a newsletter and 2 (10%) had been introduced to it by a friend or peer in recovery:

So, one of the girls that is in NA [Narcotics Anonymous] with me, she was telling me about it [the app]. Because I was saying I was struggling with my sleep...And she said to go on this app and it’ll like help you with your sleep.

Daisy, female, aged 39 years, weekly app user

Participants who had been introduced to SURE Recovery by a key worker, support worker, or professional explained that the worker had directed them to the app using a hyperlink sent in an email, by signposting them to a website, or by sending information in hard copy through the post. All participants had then successfully downloaded the app themselves. Participants who had found SURE Recovery by browsing or searching on the web mostly said that they had been trawling the webpages of a recovery organization to look for support for themselves or for others, although 1 (5%) had noticed it on social media (Instagram), and another had found it through the Apple App Store. Of the 4 participants who had learned of the app through their employment, 2 (50%) said that they had been proactively researching apps and 2 (50%) explained that details had been cascaded down to them from managers:

Part of the team I lead on is around sort of providing psychosocial interventions and group work, and...it [SURE Recovery] came up as one of the potential tools that we use with our client group.

Frank, male, aged 43 years, tried app a few times

Several participants clarified that a key factor prompting them to seek out a recovery app was the COVID-19 pandemic because this had created problems physically attending services. Some participants said that they had considered and downloaded several apps before settling on SURE Recovery, and a key reason for choosing SURE Recovery was that they thought that they might have heard of either the SURE measure or the SURE Recovery app already.

#### Personal Relevance

Although SURE Recovery had been developed for anyone in recovery or thinking about recovery from alcohol and other drug problems, our participants identified subgroups of people for whom they thought the app would be more or less appropriate. Most frequently, they suggested that the app would be more suitable for people who were in *early recovery* rather than for those who had been abstinent or stable for some time. The main reason they gave for this was that people who were in long-term recovery would be more likely to score consistently well on the SURE measure, meaning that they had little scope for improvement and therefore little incentive to return to the app to complete the measure again. In contrast, they said that someone at the start of their recovery journey would be able to complete SURE over time and see rewarding changes as their SURE scores increased on the graph:

If I was speaking to somebody that I realised was in the contemplation phase, about to begin recovery, I would say, “Look, here’s this SURE app. Jump on this, put your score in. I guarantee in a month’s time you will see that it’s having tangible benefits.” Because it’s a great way to actually show yourself and remind yourself that you’re in recovery for a reason.

Ben, male, aged 46 years, monthly app user

In addition, many participants thought that SURE Recovery would be more useful for people who had a problem with drugs, particularly heroin, rather than alcohol. This, they explained, was because the balance of the app content seemed to be on opioids, with 33% (2/6) of the features (the naloxone feature and the reading section) being very specific to heroin. Several participants stated that this made the app feel less relevant to them personally:

Well, it’s just obviously for heroin addicts, isn’t it? But that might be just because I’ve never took heroin, and I just don’t relate to it. I don’t know...Obviously people that have used heroin, it’s probably for them.

Laura, female, aged 35 years, daily app user

A small number of participants added that the emphasis on heroin was *off-putting*, and 1 (5%) reported that questions within the app on homelessness and being in prison made them doubt whether the app was really for them because they did not identify with these issues.

More generally, several participants stated that the app would be particularly relevant to people who were concerned about privacy and to those who did not like mutual aid meetings. In this regard, participants emphasized that the app provided a nonjudgmental and safe space for people to find different types of information and support without having to share their personal data. Of 20 participants, 1 (5%) added that the app could be useful for people who did not have much external support. Others felt that specific features (particularly the artwork feature, sleep tracker, or diary) might interest some individuals, and several participants had forwarded information regarding the app to peers who might (they thought) appreciate these functions.

Notwithstanding these opportunities, participants also argued that the app might be less helpful for people who were not comfortable with technology, did not have a smartphone, did not have access to mobile data, were homeless, or were using substances very heavily. In addition, some questioned whether people who were not ready to address their addiction would be interested in using the app or whether someone who was having a difficult time would be willing to engage with it:

For me, being scored [using the tracker features] works. For other people, if they go in and out of lapses, or even a full relapse, they may not want the added pressure of their scores getting worse. Because that may then...lead to further use.

Ben, male, aged 46 years, monthly app user

#### Expectations and Motivations

Most participants reported that they did not have any, or any particular, expectations before downloading SURE Recovery. Some explained that they just thought they would *give it a go* and hoped that it would offer them help or something to assist them in staying abstinent or sober. Others clarified that they did not have any *big* expectations and were simply curious. As 1 (5%) of the participants explained, addiction is *complicated* and cannot be *cured by an app*, although it is *an additional tool*. Less positively, another participant stated that they were not convinced that the app would be of much use because they preferred face-to-face meetings but had been urged to try it by their drug worker.

At the time when they downloaded SURE Recovery, some participants said that they were feeling positive and wanted to use the app to stay focused and maintain progress. In contrast, others said that they were *struggling*, *not feeling great*, or *in a terrible state* and therefore were seeking new forms of support. For example, 2 (10%) said that the app interested them because it might offer something different from Alcoholics Anonymous and Narcotics Anonymous, and another explained that they were attracted to the app because they did not find it easy to talk to people. Other participants commented that they had liked the look of the app because it seemed *easy to understand*, *simple to use*, and *always there*:

It’s something that’s there at the time, you know, when you’re having your thoughts, rather than, “Oh, you know, I’ve got to go and make an appointment to see...my counsellor”...It’s immediately there...and that’s what you need.

Amy, female, aged 43 years, weekly app user

In addition, a number of participants referred to particular features or content that had piqued their interest. Most often this was the recovery-tracking feature, which they said would enable them to monitor and reflect on their recovery journeys. However, others explained that they had been drawn by the sleep feature, and some mentioned the diary because this offered them somewhere to write down their thoughts, feelings, and activities. Several participants also reported that they had been attracted to the app because they thought that it might be able to help them with general recovery goals such as maintaining sobriety, avoiding relapse, engaging in self-help, and taking responsibility for themselves.

First impressions additionally seemed particularly important. In this regard, several participants stated that the app had seemed *different from other apps*, offered a range of content, looked as though it might provide something new, seemed to have been well researched, and was not simply about *counting* days sober. Others confirmed that it looked interesting and useful (although 1 (5%) of the participants said that they had been a little concerned that it would be too complicated for them). Some also reported that they had seen a positive review on the Apple App Store or felt that the app was *trustworthy* because it had been recommended to them by someone they respected or had been developed by a university:

You can tell from the App Store that it was developed by [name of university], and you know, like there was research being put into it. So...I think I just trusted it a bit more.

Lucy, female, aged 28 years, tried app a few times

### Engagement

#### The Appeal and Relevance of Specific Features

When participants discussed how and why they continued to engage with the app (or why they disengaged from it), the appeal and relevance of specific features were central. Most of them said that they used the recovery tracker more than any other feature. Generally, participants thought that it was motivating, interesting, useful, or fun to track their scores. Despite this, a small number of participants had not noticed the recovery tracker, and 1 (5%) had dismissed it as being too much effort to complete. In addition, a few participants thought that it was not relevant to them. This, they said, was because they scored high initially and therefore felt that they had no way of progressing, they received the same scores each time they completed it and therefore lost interest, or they thought that the questions did not apply to people such as themselves who were at a more established stage of recovery:

A lot of the questions were loaded towards like stable housing, and I think in recovery your outlook changes to the fact that what you need’s much more than that. And it [recovery tracker] didn’t go deep enough for me.

Luke, male, aged 44 years, tried app a few times

Although many participants had used the sleep tracker, some stated that they did not use it because they slept well, slept badly, or accessed other apps for sleep monitoring. The artwork feature was, meanwhile, generally appreciated, with participants variously describing it as *interesting*, *cool*, *brilliant*, and a *nice touch*. However, a few participants found it confusing and said that they did not see how it linked to the rest of the app or how people might use it if they did not have artwork to submit. The diary feature was used regularly and received some of the most positive feedback, with participants stating that they enjoyed recording their feelings (and, to a lesser extent, activities) and then looking back over their entries. Nonetheless, a few participants said that they had not used the diary feature because they did not keep a diary, preferred to record things on paper, or feared that their entries might be read by someone else, particularly if they lost their phone:

So, I suppose the aspect of the diary is [that] I would just worry somehow if I lost my phone...if somehow what you’re writing...they [diary entries] are personal and private to you.

Lucy, female, aged 28 years, tried app a few times

In contrast, the naloxone feature had not been widely used and generated quite mixed responses. A few participants appreciated having information regarding naloxone and overdosing within the app and stated that this could address misconceptions regarding overdose or would be helpful if someone witnessing an overdose panicked and forgot what to do. Nonetheless, others felt that this component of the app was not relevant to them because they were in long-term recovery or had never used heroin. The reading feature similarly evoked mixed views. Several participants said that the *Everyday Lives of Recovering Heroin Users* book did not interest them, and a participant complained that it was too long, whereas others said that they were enjoying reading it:

I just like it [Everyday Lives of Recovering Heroin Users book]...I like it just that it’s personal, it’s personal stories, it’s true, you know. It relates to obviously my life and things.

Claire, female, aged 49 years, daily app user

Most participants said that they had completed some of the research questions, with 1 (5%) emphasizing how important it is to share views and experiences with researchers to help others. Meanwhile, only a small number of participants reported that they liked the temporary COVID-19 feature, with others stating that they were tired of hearing about COVID-19 and therefore not interested in engaging with this content.

#### Perceived Benefits

Participants identified both practical and emotional benefits from using SURE Recovery, which seemed likely to maintain their interest and engagement. These benefits were reflected in both how and when people used the app. For example, some said that they used SURE Recovery when they were feeling relaxed to reflect back on their day or to help reinforce positive emotions, whereas others said that they used it when they believed that their mind might wander to drugs, were feeling concerned about their substance use, were feeling bored and needed a distraction, or thought that they might experience cravings:

I use it generally quite late at night. And I think it’s because that’s when my mind goes wandering to my sort of craving.

Liam, male, aged 33 years, weekly app user

Both practical and emotional benefits were also evident when participants discussed why they liked using particular app content and features. Thus, participants reported that the recovery tracker was useful because it enabled them to look back over their scores and see their progress, identify changes they wanted to make, and receive advice on how to advance their recovery. In addition, some stated that the feedback incentivized them to *keep going*, directed them to useful resources, was uplifting, and *gave them a boost* on a bad day. Moreover, they *enjoyed* completing the questions. One participant (5%) added that the sleep measure facilitated discussions with their physician regarding sleep, whereas others appreciated the naloxone feature because they said that it provided important information on how to save a life and made them feel more confident about responding to an overdose if needed:

With the naloxone, if somebody goes over [overdoses], it’s there ready, you know. I mean I’ve had training on naloxone, but...nobody knows how [they are] going to react when it [an overdose] happens. It’s just nice...that there’s something in your back pocket.

James, male, aged 54 years, weekly app user

Several participants additionally stated that the diary feature was valuable because it allowed them to empty their heads and put all their thoughts down in one place and it could be used as a *gratitude journal* (that is, a place to record and reflect on things for which they were thankful). Some enthused about the artwork feature and explained how this inspired them and lifted their mood, whereas others said that reading the book and learning about the experiences of others in recovery was enjoyable and could help people feel less alone. In addition, some said that the embedded links to external websites provided helpful information and a route to additional forms of assistance.

More generally, participants reported that the app was useful because it could be accessed at any time or in any place and, for some, this seemed a better option than visiting a therapist, who would need an appointment. Participants also confirmed that the app was simple to understand, did not use up much mobile data, and felt *friendly toward people who had experience of addiction*, which made them feel that they could be honest when entering their data. Equally, participants said that they appreciated the variety of content and functions and noted how not using the app for a while might alert them to an impending relapse:

And I think that’s something that’s good with the app, because you can sort of measure like the time in between using them [tracker features]. You...might look at it and think, “Oh, I haven’t been on there for ten days. Something’s not right.”

Charlotte, female, aged 43 years, daily app user

In terms of concrete benefits, several participants said that the app had brought stability to their lives and had supported them to remain stable or abstinent. However, most said that their behavior had not changed as a direct result of using the app, although a few noted that engaging with the app had been part of wider positive behavior change that they had made in their recovery. Significantly, none of the participants identified any reason why the app might be unhelpful or harmful, although 1 (5%) cautioned that it was not a replacement for other forms of support and people would likely need additional help, particularly in early recovery. A few participants also felt that there were too many questions and the app was not participative enough to be helpful as an *intervention*.

#### The Need for Improvements

Overall, there was no suggestion that the app needed to be improved in terms of usability, although a small number of participants felt that the language within the app could be simplified. Several participants also reported that they were uncertain who exactly the app was for and thought that it might be better to have a single target audience, such as people in early recovery. In addition, some participants felt that engagement with the app might increase if it had notifications and reminder features so that people would remember to complete the measures and diary each day:

Reminders as well, you know, daily reminders for people, are quite important...“What have you done today for your recovery?” That kind of stuff. I think that stuff’s pretty...important.

Luke, male, aged 44 years, tried app a few times

In terms of specific features, various participants suggested that the recovery feature could be improved by having more questions for people who were further along in their recovery, scope for scoring higher, and additional feedback on how to improve their recovery score. Other participants said that they would have liked more feedback on how to improve their sleep score and felt that the inclusion of meditation and relaxation aids would be useful additions. No particular improvements were suggested to the artwork feature or diary content, other than a passcode to increase the diary’s security and privacy. Meanwhile, participants who thought that the app was too opiate focused expressed a desire for more reading and resources on other substances, such as a book on recovery from alcohol problems.

Turning to new features, many participants wanted to see a simple sobriety tracker that recorded an individual’s number of days abstinent, whereas others recommended the inclusion of affirmations (that is, positive statements that can help people overcome negative and self-sabotaging thoughts). Participants additionally suggested including a section on other elements of well-being, such as nutrition, exercise, and mental health or mood. Finally, some thought that the app would be better if it enabled them to connect with, and talk to, others in recovery; for example, through a live chat or newsfeed or by having opportunities to submit personal stories and experiences:

I feel there should be like maybe where you can sign up and you can interact with other people in recovery. So that, you know, you can kind of support each other, and also meet other people that are sober and in recovery.

Liam, male, aged 33 years, weekly app user

## Discussion

### Principal Findings

Our analyses identified 3 main factors influencing uptake of, and 3 main factors influencing engagement with, the SURE Recovery app. Importantly, however, there were similarities and overlap between the uptake and engagement factors. In terms of uptake, study participants learned about the app through various sources but seemed particularly likely to download it if it came to their attention through people, services, or social media they trusted. Uptake also seemed to increase when people did not want, or were unable, to access more formal support. In addition, SURE Recovery was deemed more suitable for people who were in early recovery and users of opioids, as well as people who had access to, and a level of understanding of, technology and a degree of motivation for recovery. Although overall expectations of SURE Recovery tended to be low, particular app features appeared to pique interest and draw users in initially.

In terms of engagement, participants particularly liked the recovery and sleep trackers and the diary. The embedded research questions were considered acceptable, but the opioid-specific material (the naloxone section and, to a lesser extent, the book) were more controversial and seemed to be associated with a degree of disengagement by some. Participants attributed a range of practical and emotional benefits to using SURE Recovery, with no reports of any harm caused. Benefits included easy access to useful information, support in maintaining stability and abstinence, reinforcement of positive behavior changes, enjoyment, increased motivation to recover, and improved mood. Despite this, participants noted that the app was not a standalone *intervention* that could *cure addiction*, and some wanted greater clarity regarding the intended audience. In addition, participants recommended a range of new features, including notifications and reminders, more content on alcohol and other substances, a simple method for counting days sober, and opportunities for real-time social interaction with other people in recovery.

In practice, our research findings replicated some of the early insights we had gained from the first *discover* stage of co-designing SURE Recovery. During this initial developmental work, interview and focus group participants had also stated that they valued different types of formal and informal support, enjoyed connecting with others in similar situations, felt that keeping a log of their recovery was helpful, wanted advice on the types of support available, liked tracking features, and desired inspirational quotations. This high level of concordance between the 2 stages of our work seems to validate our decision to adopt a user-focused design process because the end users clearly appreciated the features recommended to us by people with experience of substance use during the developmental stage. This finding differs from the conclusion of a systematic review of health and well-being smartphone app uptake and engagement conducted by Szinay et al [11] that reported that study participants who discussed a hypothetical app did not always agree with those who gave their views after actually using an app. Such inconsistency with our findings merits further investigation because the good concordance we identified clearly suggests that soliciting and incorporating end-user views into app design can improve uptake and engagement later [30].

Otherwise, many of our findings were broadly consistent with both the review by Szinay et al [11] and a range of other published literature. As reported by Szinay et al [11], we found that when people were recommended the app, uptake increased; the provision of health information, reminders, self-monitoring, positive tone, social networking, and perceived utility were linked to better engagement; and app literacy skills affected both uptake and engagement. Equally, we identified support for various items of the Mobile App Rating Scale; for example, our participants appreciated fun, interest, interactivity, usability, information quality, suitability for the target audience, and credibility [31]. In line with other studies [2,7,8], we established that people liked convenience and privacy, a nonjudgmental tone, and the opportunity to see scores and monitor personal progress. More negatively, meanwhile, our analyses confirmed that uptake and engagement were likely to be undermined by poor access to mobile technology, Wi-Fi, or mobile data, and low user motivation for behavior change [9,11,14].

Importantly, our findings also yielded newer insights. First, participants were attracted to SURE Recovery based on first impressions, including the hope that the app would offer them something useful, different, or new; it looked interesting; and it seemed trustworthy because it was developed by a university or had been recommended to them. Second, participants approached SURE Recovery with very modest expectations. Indeed, they were willing to download it based on their need for support or because they were curious. Consequently, we had no need to promote, advertise, or market SURE Recovery using promises that it would *stop addiction*, *cure cravings*, or *change lives*. Third, participants indicated that a key factor in maintaining engagement was how the app made users feel. For example, our participants said that they valued the enjoyment gained from using the app, they felt motivated and heartened when they saw their scores or looked at the artwork, they were able to clear their heads when writing things down in the diary, and they experienced a connection to others when reading the book. Fourth, our analyses highlighted the significance of relatability; thus, participants seemed unlikely to download or engage with the app if they could not relate to its content, especially if that content undermined their sense of identity (for example, by inaccurately implying that they used heroin, were homeless, or committed crimes).

Taken together, our findings and reflections have potential relevance for other researchers and app developers. For example, we believe that our co-design process was critical in ensuring that SURE Recovery is user friendly, easy to understand, motivating, and trustworthy, and we recommend this collaborative way of working to others [8,30]. Nonetheless, we developed SURE Recovery in response to user demand with limited consideration of our precise target audience and whether we should be including empirically based behavior change techniques [2,9,15,16]. With hindsight, we might have been wiser to have chosen a more focused audience (such as people using heroin or people in early recovery) and then developed and disseminated the app in response to their particular wishes. Likewise, we might have included additional evidence-based behavior change techniques such as goal setting, action planning, and social support [32-34]. These potential changes notwithstanding, we would still have retained our co-design process, given that even very powerful behavior change techniques are undermined if they are delivered in a way that people do not understand, do not trust, or deem boring or unacceptable.

Given the importance of personal and trusted recommendation in relation to uptake, we conclude that an effective dissemination strategy requires a network of supportive partners, that is, credible organizations and individuals who will champion the app by telling others that it exists, linking it to their own websites and informational materials, and proactively disseminating it by means of social media. In addition, it would be helpful if these partners were able to offer guidance and support to people who might use the app to ensure that they know how to download it, understand all the functions, and are able to capitalize on what is being offered [11]. More generally, our findings remind us that apps need to be maintained and regularly updated in response to user feedback. This requires resources (money, time, and expertise) alongside a business model for sustainability [1]. In addition, consideration needs to be given to *competitor apps*. Some of our participants stated that there were other apps for sleep; therefore, they were not interested in using SUSS. Over time, we will likely see more free recovery apps published, some of which will probably have additional capacities (for example, GPS, motion sensors, biophysical monitoring, 24-hour professional support, or linkages to primary care–based treatment) [1,7,15]. Although we appreciate that some potential app users may feel overwhelmed or confused by having too much choice, this is unlikely to be a problem with respect to recovery apps aimed at people experiencing alcohol or other drug problems where options are currently very limited. We therefore reject the view that *competitor apps* are a problem. Instead, we feel that having more well-designed apps that seek to support people in overcoming problems with their substance use indicates that this is a viable space for technological innovation and the availability of a pool of apps should provide welcome choice for an often-underserved population.

Turning finally to how our findings have started to shape and influence our own work, we have recently begun to develop and support a community of SURE Recovery users. To this end, we have recruited a small group of people (SURE Recovery champions) who have lived experience of substance use, have good information technology skills, and work or volunteer in addiction services in different areas of the United Kingdom. These individuals have been provided with a tablet computer, training in how to use and explain SURE Recovery to others, a small honorarium, and out-of-pocket expenses to enable them to travel to services and demonstrate the app. The champions also meet monthly on the web with members of the core app development team to share knowledge and understanding. This initiative has been established to capitalize on our finding that people are more interested in the app when they hear about it from a trusted source. In addition, by connecting the tablet to the free Wi-Fi within services, the SURE Recovery champions increase the app’s accessibility to people who may not have hardware or their own mobile data plans.

Beyond this, the dynamic *hot topic of the month* feature introduced in March 2021 has enabled us to begin to balance out the content of SURE Recovery by adding more questions and resources relating to alcohol and wider topics that are not opioid specific (such as mental health, diet and eating, peer support, mindfulness, and stigma). We have also used the hot topic feature to invite app users to provide us with *words of wisdom* to pass on to other people in recovery, and some of these reflections have now been included in the banner of the app. This responds to requests to include inspirational quotations, while also helping to make the app more participative and increasing the feeling of community among users. To supplement this, we have amplified our social media presence with a Facebook page, YouTube channel, Twitter handle, and Instagram account. Going forward, we will also be meeting again with our app developer to discuss the inclusion of push notifications and reminders, as well as brainstorming other ideas for updating the app based on our research findings.

### Limitations

Our study and analyses inevitably include limitations. The research was conducted by members of the team who developed SURE Recovery. Our findings may consequently suffer from social desirability bias [35] because the people who were interviewed might not have been as critical as they would have been if the research team had been wholly independent of the app. Equally, we only interviewed 20 people. Although we were careful in selecting participants who reported different levels of engagement with SURE Recovery, we recognize that those interviewed were not necessarily representative of people downloading or using our app. Furthermore, people who download and use SURE Recovery are not representative of all people using substances. This is clear from the fact that our interview participants were all aged 25-63 years and identified as White British. That our findings do not capture the views and experiences of people of color is a particular shortcoming within a field that has historically underrepresented populations identifying as non-White. We hope that others developing and evaluating addiction-related apps will learn from this limitation and will consider collecting and analyzing ethnicity data to better understand if and why some populations may not engage and to help ensure that future apps are clearly relevant to a range of ethnic and racial groups. Because of these limitations, we cannot claim that our findings reflect the views and experiences of people with different ethnicities and demographic characteristics. Nonetheless, we are encouraged by the fact that key patterns and themes identified in our data are found in other research. This provides a degree of reassurance that our findings, although not empirically generalizable, are likely to be relevant and *transfer* to other related apps and settings [27,28].

### Conclusions

To conclude, we return to the question of whether there is a genuine future for mHealth apps aimed at people in recovery from alcohol and other drug problems and, if so, what can be done to promote uptake and engagement. Our findings are cautiously positive but show that additional effort is needed. Although first impressions, trusted recommendations, personal relevance, and perceived benefits will all play a role, addiction recovery app uptake and engagement continue to be undermined by broader structural issues of digital exclusion and marginalization [36,37]. Until there is wider access to devices and mobile data and better universal information technology literacy, the potential of any digital intervention is not likely to be achieved [14]. For the foreseeable future, we will therefore need addiction service providers to support mHealth interventions by providing access to devices, onsite Wi-Fi, and training and support in using digital technology. Meanwhile, people who do use apps will not always be expecting them to reduce their substance use or increase their days sober. They may also download and engage with an app because it looks interesting, makes them feel better, lifts their mood, helps them to feel connected with others, or is fun. Accordingly, we should not assess the success of addiction recovery apps based only on objective measures of changes in substance use. As an app can only ever be a cog in a wider ecosystem of support and treatment, we also need to judge its impact through more subjective indicators of health and well-being that may be transient and difficult to quantify but important nonetheless.

## Abbreviations

mHealth: mobile health
SURE: Substance Use Recovery Evaluator
SUSS: Substance Use Sleep Scale
